# Identifying the intervention mechanisms of polydatin in hyperuricemia model rats by using UHPLC-Q-Exactive Orbitrap mass spectroscopy metabonomic approach

**DOI:** 10.3389/fnut.2023.1117460

**Published:** 2023-04-28

**Authors:** Xueli Ge, Zhenguo Su, Yuhao Wang, Xue Zhao, Kaifei Hou, Shuna Zheng, Pengjiao Zeng, Zhongqi Shi, Senhao Hu, Yuqing Wang, Mengchen Zhou, Jiayu Zhang, Xiulian Li

**Affiliations:** ^1^School of Pharmacy, Binzhou Medical University, Yantai, China; ^2^Affiliated Hospital of Binzhou Medical College, Yantai, China; ^3^Medical Research Center, Affiliated Hospital of Qingdao University, Qingdao, Shandong, China

**Keywords:** polydatin, urate-lowing effects, metabolomics, mechanism, UHPLC-Q-Exactive Orbitrap

## Abstract

**Introduction:**

Polydatin is a biologically active compound found in mulberries, grapes, and *Polygonum cuspidatum*, and it has uric acid-lowering effects. However, its urate-lowering effects and the molecular mechanisms underlying its function require further study.

**Methods:**

In this study, a hyperuricemic rat model was established to assess the effects of polydatin on uric acid levels. The body weight, serum biochemical indicators, and histopathological parameters of the rats were evaluated. A UHPLC-Q-Exactive Orbitrap mass spectrometry-based metabolomics approach was applied to explore the potential mechanisms of action after polydatin treatment.

**Results:**

The results showed a trend of recovery in biochemical indicators after polydatin administration. In addition, polydatin could alleviate damage to the liver and kidneys. Untargeted metabolomics analysis revealed clear differences between hyperuricemic rats and the control group. Fourteen potential biomarkers were identified in the model group using principal component analysis and orthogonal partial least squares discriminant analysis. These differential metabolites are involved in amino acid, lipid, and energy metabolism. Of all the metabolites, the levels of L-phenylalanine, L-leucine, *O*-butanoylcarnitine, and dihydroxyacetone phosphate decreased, and the levels of L-tyrosine, sphinganine, and phytosphingosine significantly increased in hyperuricemic rats. After the administration of polydatin, the 14 differential metabolites could be inverted to varying degrees by regulating the perturbed metabolic pathway.

**Conclusion:**

This study has the potential to enhance our understanding of the mechanisms of hyperuricemia and demonstrate that polydatin is a promising potential adjuvant for lowering uric acid levels and alleviating hyperuricemia-related diseases.

## 1. Introduction

Hyperuricemia (HUA) is a metabolic disease caused by the accumulation of excess uric acid (UA) in the serum due to abnormalities in purine metabolism or UA excretion (1). Along with the changes in diet and lifestyle, the prevalence of HUA has been increasing and mostly affects young people (2, 3). Overproduction of UA in the body can cause a series of complications, such as gout (4), diabetes, cardiovascular disease, and chronic kidney disease (5). Currently, the main therapy for HUA is urate reduction *via* the inhibition of UA production or acceleration of UA excretion. Effective drugs commonly exert rapid effects. However, the alleviation of symptoms is usually short-lived and recurs without continued drug treatment, which causes unwanted side effects (6–8). Therefore, it is necessary to develop alternative compounds that are more effective and less toxic for managing HUA.

In recent years, plant-derived natural compounds have been recognized as efficacious and largely benign and have found wide acceptance as medicines or lead compounds globally (9). Polydatin is a polyphenolic monomer compound abundant in mulberries, grapes, and *Polygonum cuspidatum* and has been shown to have a wide variety of bioactivities, such as liver and kidney protection, anti-inflammatory, antioxidant, and antitumor effects (10, 11). Many studies have demonstrated that polydatin has urate-lowering effects by inhibiting xanthine oxidase activity, reducing the UA synthesis rate, and downregulating mURAT1, mGLUT9, and mABCG2 expression to promote UA excretion in renal tissues (12, 13). In addition, polydatin mitigates kidney injury by inhibiting inflammasome activation (14). However, the underlying intervention mechanism of polydatin in lowering urate is complex and requires further investigation.

Small-molecule metabolites directly reflect pathological processes following external stimuli or disturbances. Alterations in endogenous metabolites have recently been used to study disease pathophysiology and evaluate the toxic and therapeutic effects of drugs (15, 16). Metabolomics provides a method for detecting and analyzing the types, quantities, and varying patterns of endogenous small-molecule metabolites (amino acids, lipids, sugars, etc.) from biological samples in a timely and comprehensive manner (17). Metabolomics has been successfully employed to screen potential biomarkers, characterize physiological or pathological conditions in various diseases, and evaluate metabolic pathway disorders (18–20). However, few studies have described the urate-lowering mechanisms of polydatin, focusing on endogenous small-molecule substances in animal experiments.

In the present study, we employed a metabolomics approach using UHPLC-Q-Exactive Orbitrap mass spectrometry (MS) to explore the mechanism underlying the urate-lowering effects of polydatin in rats. A rat model of potassium oxonate-induced HUA was established to analyze multiple targets, including a low concentration of UA, protection of kidney and renal function, and regulation of blood lipid levels to evaluate the amelioration of HUA by polydatin. Moreover, we screened potential biomarkers in serum samples to assess the therapeutic efficacy of polydatin. The regulation of the metabolic network by polydatin was illustrated using metabolic pathway analysis. This study elucidated the potential mechanisms of polydatin in moderating HUA and provided alternative prevention and treatment options for HUA.

## 2. Materials and methods

### 2.1. Chemical and reagents

Potassium oxonate was purchased from Sigma–Aldrich (St. Louis, MO, USA). Benzbromarone and polydatin (≥95% purity) were purchased from Yuan-Ye Biotechnology Technology (Shanghai, China). Carboxymethyl cellulose sodium was purchased from Aladdin (Shanghai, China). D-Fructose was obtained from Solarbio Technology (Beijing, China). HPLC-grade formic acid and acetonitrile were supplied by Fisher (Waltham, MA, USA). Deionized water was obtained using a Milli-Q system (Merck, USA).

### 2.2. Animals

A total of 32 male Sprague Dawley (SD) rats (200 ± 20 g) were purchased from Jinan Pengyue Laboratory Animal Breeding (License No: scxk (Ru) 20190003). All experimental protocols were approved by the Animal Ethics Committee of Binzhou Medical University (No. 2022-353).

### 2.3. Establishment and treatments in a rat model

The SD rats were acclimated for 3 days prior to the experiments (24 ± 2°C and a 12/12 h light/dark cycle). The rats were randomly divided into four groups: (1) a control group, a model group, a positive group, and a polydatin group, with eight rats in each group. Potassium oxonate (300 mg/kg) and 10% fructose water were used to establish a hyperuricemic rat model (21). Potassium oxonate was resuspended in a 0.5% sodium carboxymethylcellulose (CMC-Na) solution and administered by gavage to rats in the model, positive, and polydatin groups at 8:00 every morning. After 1 h, the rats in the positive group were intragastrically administered 20 mg/kg benzbromarone, and the rats in the polydatin group were orally administered 50 mg/kg benzbromarone for 28 consecutive days. The doses of polydatin were determined based on previous reports (14, 22, 23). Rats in the control group were administered the same volume of 0.5% CMC-Na water by gavage. Benzbromarone and polydatin were dissolved in 0.5% CMC-Na. During the experiment, the rats in the positive and polydatin groups were fed 10% fructose water.

### 2.4. Sample collection and preparation

All rats were anesthetized by intraperitoneal injection of chloral hydrate (30 mg/kg) after fasting for 24 h. Abdominal aortic blood samples were collected and centrifuged at 3,500 rpm for 15 min at 4°C, and the supernatants were collected. Serum (200 μl) was used to determine biochemical parameters using an automatic biochemical analyzer, and the remaining serum samples were stored at −80°C for metabolomics analysis.

Intact liver and kidney tissues were removed and washed with pre-cooled saline, followed by drying with filter paper. Fresh tissues were fixed in 4% paraformaldehyde for more than 24 h, trimmed, embedded in paraffin, routinely sectioned, dewaxed in xylene, dehydrated in gradient ethanol, stained with hematoxylin for 3–5 min, washed, stained with eosin for 5 min, washed, dehydrated in gradient ethanol, made transparent in xylene, and sealed with neutral glue (24). Finally, the pathological states of the liver and kidneys were observed under a light microscope.

### 2.5. Sample preparation for a metabolomic study

The serum sample was thawed at 4°C. Acetonitrile (400 μl) was added to 100 μl of a serum sample for protein precipitation. After vortexing for 1 min, the samples were centrifuged at 14,000 rpm for 10 min at 4°C. Subsequently, 150 μl of the supernatant was separated and evaporated to dryness with nitrogen at 27°C. The residue was dissolved in 100 μl of an 80% (v/v) acetonitrile aqueous solution and then injected for UHPLC-Q-Exactive Orbitrap MS analysis. Quality control (QC) samples were prepared in the same manner as described above and were mixed with all samples in equal volumes (10 μl).

### 2.6. UHPLC-Q-Exactive Orbitrap MS analysis

Liquid chromatography was performed using a Q-Exactive Focus Orbitrap MS (Thermo Electron, Bremen, Germany) connected to a Thermo Scientific Dionex Ultimate 3000 RS (Thermo Fisher Scientific, CA, USA) equipped with an ACQUITY UPLC BEH C18 column (2.1 mm × 100 mm, 1.7 μm, Waters Corp., USA). The flow rate was 0.28 mL/min, and the column temperature was maintained at 45^°^C. The mobile phases were 0.1% formic acid in water (solvent C) and acetonitrile (solvent D). The gradient elution program was as follows: 0–1 min, 95% C; 1–5 min, 95–55% C; 5–10 min, 55–35% C; 10–15 min, 35–20% C; 15–15.1 min, 20–5% C; 15.1–17 min, 5% C; 17–17.1 min, 5–95% C; 17.1–20 min, 95% C. The injection volume was 2 μl.

The electrospray ionization (ESI) source was operated in both positive and negative ion models for MS analysis. The following operating parameters were used: capillary voltage of 35 V; capillary temperature of 320°C; the tube lens voltage of 110 V; auxiliary gas flow rate of 10 arb. The m/z range was set at 100–1,000 Da.

### 2.7. Data analysis and identification of potential biomarkers

All data acquired from UHPLC-Q-Exactive Orbitrap MS were normalized using Compound Discover 3.0 (Thermo Fisher) software for pre-processing (peak identification, peak matching, data alignment, and experimental grouping design). Retention times and MS fragments were generated by analysis. Subsequently, the obtained data matrices were analyzed by principal component analysis (PCA) and orthogonal partial least squares discrimination analysis (OPLS-DA) using SIMCA-P 13.0 (Umetrics, Umeå, Sweden) (25–27). Metabolites with significant differences between different groups were screened based on variable importance in the projection (VIP) values (VIP>1.0) and Student’s *t*-test (*p* < 0.05). The structural identification of differential metabolites was conducted using the HMDB^1^ (and PubChem databases (28–31).^2^

### 2.8. Pathway analysis

Metabolic pathway analysis was performed using MetaboAnalyst.^3^ Biochemical interpretation of the metabolic pathways was performed using the KEGG database^4^ and relevant references.

## 3. Results

### 3.1. Effects of polydatin on weight and serum biochemical indicators in hyperuricemic rats

**FIGURE 1:**
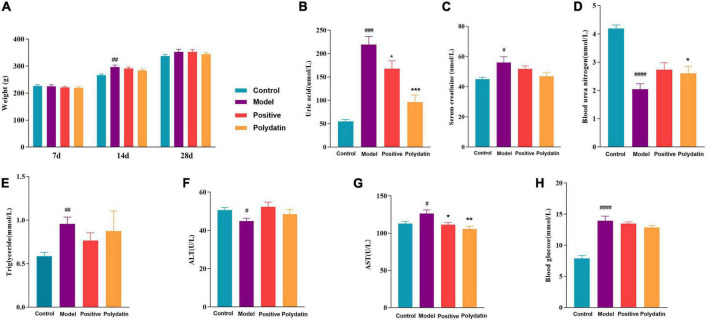
Effects of polydatin on biochemical indicators in serum from HUA rats. **(A)** Weight change of rats in 28 days. **(B)** Serum uric acid (SUA). **(C)** Serum creatinine (Scr). **(D)** Blood urea nitrogen (BUN). **(E)** Serum triglyceride (TG). **(F)** Alanine transaminase (ALT). **(G)** Aspartate aminotransferase (AST). **(H)** Blood glucose. Values are given as the mean ± SEM (*n* = 8) (*p < 0.05, ^**^p < 0.01, ^***^p < 0.001, vs. model group; ^#^*p* < 0.05, ^##^*p* < 0.01, ^###^*p* < 0.001, ^####^*p* < 0.0001, vs. control group).

An increased serum uric acid (SUA) level is considered a typical indicator of HUA. Blood urea nitrogen (BUN) and serum creatinine (Scr) are the final nitrogenous products of protein metabolism, and their levels indicate impaired or normal renal function. Alanine transaminase (ALT) and aspartate aminotransferase (AST) are valuable markers of liver function. As shown in Figure 1A, the changes in rats’ body weight were insignificant, implying that the dose of polydatin used in the study was safe for HUA rats. Compared to the control group, at 28 days, the levels of SUA, Scr, AST, TG, and blood glucose were significantly higher, and the levels of BUN and ALT were significantly lower in the model group at 28 days (*p* < 0.05). After administering polydatin and benzbromarone as positive controls, there was a recovery trend in SUA, BUN, AST, TG, blood glucose, Scr, and ALT levels. This indicated that polydatin could have a urate-lowering effect in a rat model.

### 3.2. Effects of polydatin on liver and renal injury in hyperuricemic rats

**FIGURE 2:**
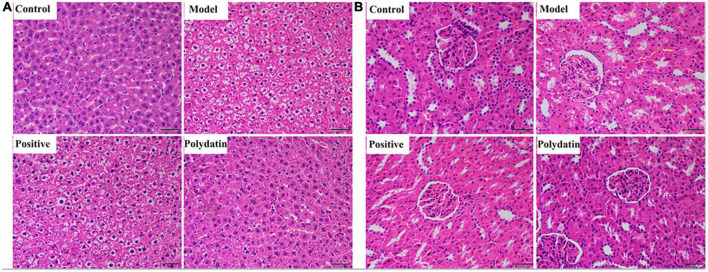
Histopathological evaluation of rat liver **(A)** and kidney **(B)** in different treatment groups. Scale bar = 100 μm.

Histopathological hallmarks of liver cells were vacuolar degeneration, local hepatocyte necrosis, and hepatic cords arranged less neatly in the liver of hyperuricemic rats compared with those in normal rats. These pathological states were attenuated when treated with polydatin (Figure 2A). In addition, the histological analysis showed significant pathological changes in the kidneys of model rats. Noticeable pathological changes in renal tubular epithelial cells included atrophy of the glomerulus and vacuolar degeneration compared with the control group. As was observed for the pathological states in the liver, those in the kidneys were also ameliorated by treatment with polydatin (Figure 2B). The results indicated that the injuries induced by high UA levels were improved to varying degrees by polydatin. The histological results were in accordance with the levels of UA observed above in hyperuricemic rats.

### 3.3. Serum metabolic analysis

To systematically describe the underlying mechanisms of polydatin-ameliorating HUA, UHPLC-Q-Exactive Orbitrap MS was used to analyze serum samples from rats in positive and negative ion modes. Representative serum base peak ions (BPI) of serum samples from the control, model, positive, and polydatin groups were obtained (Supplementary Figure 1). All peaks in the serum samples from each group of rats were well separated at 20 min in both ESI^+^ and ESI^–^ modes. The metabolic profiles of each group were different, indicating changes in the endogenous metabolic profiles between different groups.

**FIGURE 3:**
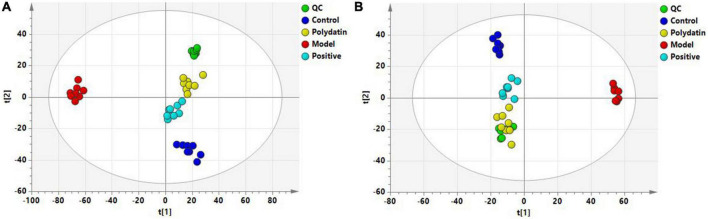
PCA score plots of QC and four groups based on serum metabolic profiles **(A)** ESI^+^ mode; **(B)** ESI^–^ mode.

Principal component analysis score plots were used to understand the differences in the serum metabolic profiles of rats in each group. We noticed a clear separation trend in the different groups in both ESI^+^ and ESI^–^ mode experiments, suggesting that endogenous metabolites differed significantly among the control, model, positive, and polydatin groups, and the group difference was more evident than the individual difference (Figure 3). In addition, the QC samples aggregated significantly, indicating good repeatability and stability (Figures 3A, B).

### 3.4. Identification of potential biomarkers

**FIGURE 4:**
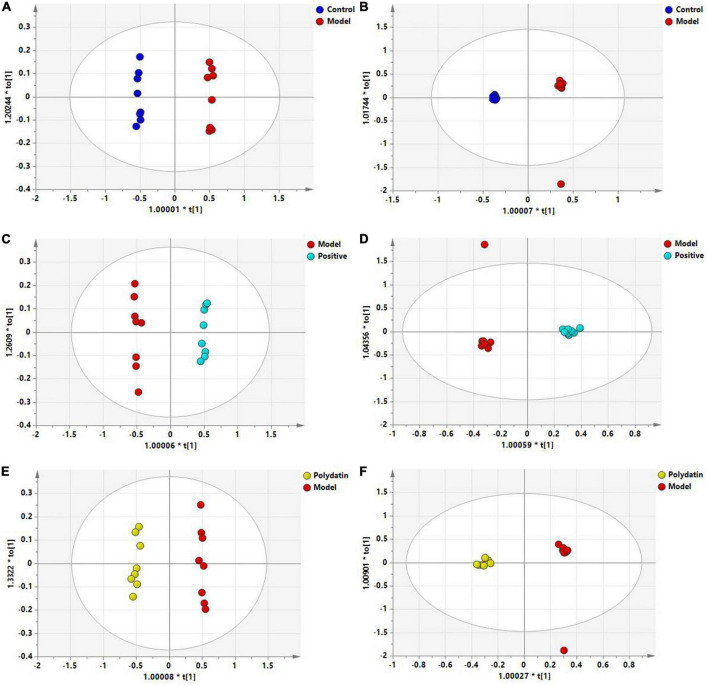
OPLS-DA score plots of serum samples in different groups. Control and model groups in ESI^+^ mode **(A)** and ESI- mode **(B)**; positive and model groups in ESI^+^ mode **(C)** and ESI^–^ mode **(D)**; polydatin and model groups in ESI^+^ mode **(E)** and ESI^–^ mode **(F)**.

To maximize class differentiation, supervised OPLS-DA was applied to differentiate among the control, model, positive, and polydatin groups to obtain potential biomarkers to evaluate the therapeutic effects of polydatin. As shown in Figures 4A, B, the control and model groups were clearly separated into different areas in both positive and negative ion modes, indicating significant metabolic changes in the HUA rat model. The R^2^Y and Q^2^ parameters were used to evaluate the OPLS-DA model. R^2^Y and Q^2^ were 0.986 and 0.982, respectively, in ESI^+^ mode, and 0.998 and 0.987, respectively, in ESI^–^ mode (Supplementary Table 1). These values were close to 1, suggesting that the model was in good agreement with the experiment data. The model was validated by 200 rounds of permutation tests (Supplementary Figure 2A, B), and all R^2^ or Q^2^ values on the left were reliable and not over-fitted. To assess the urate-lowering effects of polydatin, the serum metabolic profiles of the positive and polydatin groups were compared with those of the model based on the OPLS-DA model (Figures 4C–F), and the scatter plots were significantly separated. Meanwhile, R^2^Y and Q^2^ (Supplementary Table 1) and permutation tests (Supplementary Figures 2C–F) confirmed that the models were successful. These results demonstrated that polydatin exhibits urate-lowering properties.

The S-plot (Supplementary Figure 3) was derived from the OPLS-DA, and each spot represented a substance that was used to screen endogenous biomarkers by detecting high contributions and correlations. Of all the metabolites, only those with VIP>1 and *p* < 0.05 by *t*-test were identified as potential biomarkers between the control and model groups. The structural information of the metabolites, such as molecular weight and MS/MS fragmentation, was confirmed using freely accessible public databases (HMDB and KEGG). According to the criteria above, potential biomarkers were chosen and identified between the control and model groups (Table 1). A total of 14 endogenous altered metabolites were identified in the serum of the model rats compared to that of the control group, related to lipid and amino acid metabolism and other metabolic pathways. The identified biomarkers are summarized in Table 1.

**TABLE 1:** Identification of different metabolites and the change trends of metabolites in different groups.

No.	Metabolites	Formula	Rt (min)	ESI mode	Theoretical mass m/z	Experimental mass m/z	Error (ppm)	VIP score	MS^n^	Trend		
										**Model**	**Positive**	**Polydatin**
1	Alpha-methylstyrene	C_9_H_10_	9.69	[M+H]^+^	119.0853	119.0851	−3.58	2.29	103,92,78	↓^####^	↑****	↑****
2	L-Leucine	C_6_H_13_NO_2_	1.45	[M+H]^+^	132.1017	132.1015	−2.91	3.51	87,86,56	↓^###^	↑****	↑****
3	3-Phenyl-2-propen-1-ol	C_9_H_10_O	9.69	[M+H]^+^	135.0800	135.0798	−4.60	1.30	108,54	↓^####^	↑****	↑****
4	Nona-2,6-dienal	C_9_H_14_O	19.99	[M+H]^+^	139.1114	139.1112	−3.39	1.35	83,69,56	↓^####^	—	↑****
5	L-Phenylalanine	C_9_H_11_NO_2_	2.07	[M+H]^+^	166.0858	166.0856	−3.82	2.33	149,120,91	↓^###^	↑****	↑****
6	Dihydroxyacetone phosphate	C_3_H_7_O_6_P	19.12	[M+H]^+^	171.0052	171.0050	−1.64	1.40	97,89,72	↓^####^	↑****	↑****
7	L-Tyrosine	C_9_H_11_NO_3_	1.16	[M+H]^+^	182.0807	182.0805	−3.40	1.81	165,154,136	↑^###^	—	—
8	O-Butanoylcarnitine	C_11_H_21_NO_4_	3.28	[M+H]^+^	232.1537	232.1534	−3.63	1.35	173,144,86,57	↓^###^	↑**	↑****
9	Mono-(2-ethylhexyl)phthalate	C_16_H_22_O_4_	19.99	[M+H]^+^	279.1584	279.1580	−3.71	1.30	164,112,99,57	↓^####^	↑****	↑****
10	Sphinganine	C_18_H_39_NO_2_	10.09	[M+H]^+^	302.3045	302.3041	−4.02	11.91	284,85,71,57	↑^####^	↓****	↓****
11	Phytosphingosine	C_18_H_39_NO_3_	8.53	[M+H]^+^	318.2994	318.2990	−3.77	15.82	155,85,71,57	↑^####^	↓****	↓****
12	Valerenic acid	C_15_H_21_O_2_	11.61	[M-H]^–^	233.1545	233.1545	1.48	1.18	193,165,121,110	↑^###^	↓****	—
13	6-Gingerol	C_17_H_26_O_4_	8.74	[M-H]^–^	293.1762	293.1788	1.08	2.69	237,222,210,142	↓^####^	↑****	↑****
14	1,4-Bis (2-ethylhexyl) sulfosuccinate	C_20_H_37_O_7_S	13.73	[M-H]^–^	421.2271	421.2272	1.54	1.95	283,227,80	↓^####^	↑****	↑****

**p < 0.01, ****p < 0.0001, compared with model group; ^###^p < 0.001, ^####^*p* < 0.0001, compared with control group. ^–^Denotes no statistically significant difference.

### 3.5. Metabolic pathway analysis of identified biomarkers

#### 3.5.1. Alteration in metabolic pathways in hyperuricemic rats

**FIGURE 5:**
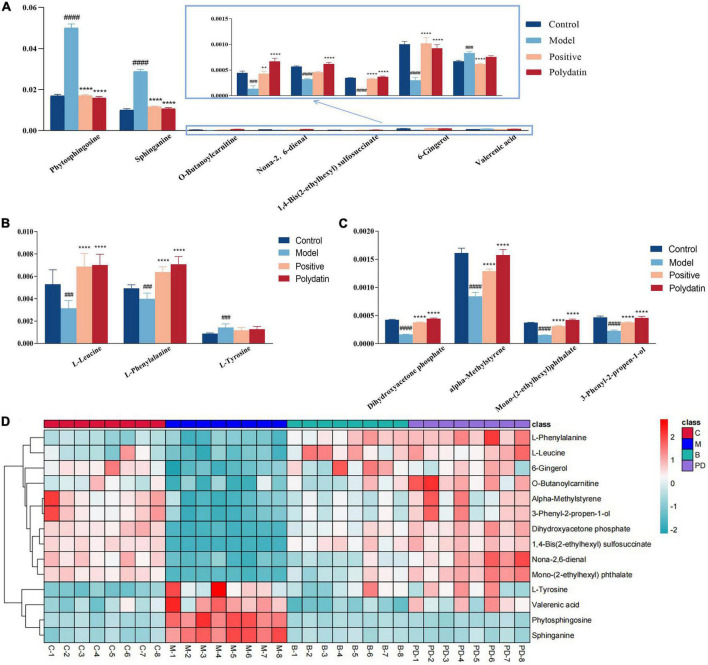
The relative levels of differential metabolites in serum from control group, model group, positive group and polydatin group. Comparison of signal intensity of significant metabolites related with lipid metabolism **(A)**, amino acid metabolism **(B)** and other metabolism **(C)** in different groups **(D)**. The hierarchical clustering heatmap of differential metabolites in serum from different groups. Deeper red represents up-regulation and deeper blue represents down-regulation, and each cell stands for one metabolite (**p < 0.01, *⁣*⁣**p < 0.0001, vs. model group; ^###^*p* < 0.001, ^####^*p* < 0.0001, vs. control group).

**FIGURE 6:**
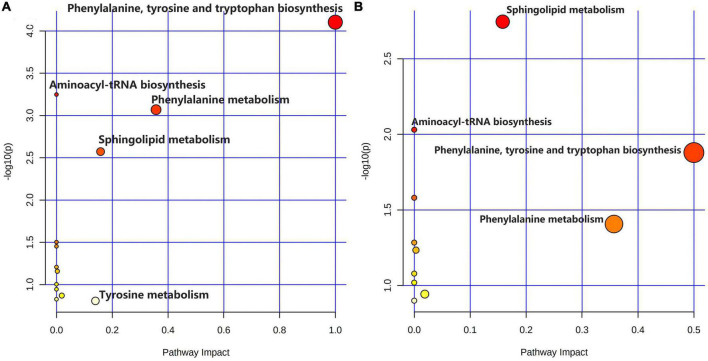
Summary of metabolic pathway analysis. **(A)** Control group vs. model group; **(B)** model group vs. polydatin group.

As shown in Figures 5A–C, comparing the signal intensity of differential metabolites, 14 metabolites changed significantly in the model group compared to that in the control group. The levels of L-tyrosine, sphinganine, phytosphingosine, and valerenic acid increased in the model group, whereas the levels of alpha-methylstyrene, L-leucine, L-phenylalanine, 3-phenyl-2-propen-1-ol, nona-2,6-dienal, dihydroxyacetone phosphate, *O*-butanoylcarnitine, mono-(2-ethylhexyl) phthalate, 6-gingerol and 1,4-bis (2-ethylhexyl) sulfosuccinate were decreased in the model rats. To investigate the metabolic pathways in hyperuricemic rats, metabolites with apparent alterations were imported into MetaboAnalyst 5.0. The metabolic networks were mainly involved in phenylalanine, tyrosine, and tryptophan biosynthesis, phenylalanine metabolism, sphingolipid metabolism, aminoacyl-tRNA biosynthesis, and tyrosine metabolism in rats treated with potassium oxonate (Figure 6A).

#### 3.5.2. Alteration in metabolic pathways in hyperuricemic rats treated with benzbromarone and polydatin

The heatmap (Figure 5D) represents the changes in 14 altered metabolites in the four groups. Figure 5D shows the information of metabolites as the ordinate and the information of groups as the abscissa. Each cell represents one metabolite, and deep red and deep blue represent upregulation and downregulation, respectively. The results showed that 14 metabolites varied significantly in the model group, and the levels of the endogenous differential metabolites were reversed in hyperuricemic rats after the administration of benzbromarone and polydatin. Of all the differential metabolites, 12 metabolites associated with HUA were reversed significantly by polydatin and had a tendency to return to the levels of the normal group (Figures 5A–C). Furthermore, metabolic network analysis comprehensively demonstrated that polydatin interfered with pathways in the hyperuricemia group (Figure 6B). Taken together, the above results demonstrate that polydatin is an effective urate-lowering therapy.

## 4. Discussion

Sustaining high serum and UA levels can contribute to serious diseases. However, UA concentration is the only diagnostic indicator for HUA, and there are no satisfactory ways to reduce UA levels without harmful side effects. Polydatin (3,4,5-trihydroxystilbene-3-β-D-glucoside) is a natural resveratrol glucoside derived from plants. Containing three phenolic hydroxyl groups, it acts as an oxygen radical scavenger.

Polydatin is found in *Polygonum cuspidatum*, grapes, red wine, peanuts, and many other commonly consumed foods. Several studies have suggested that polydatin has many pharmacological activities, including effectively decreasing UA levels (10). In this study, we explored the interventional effects of polydatin in an HUA rat model using the UHPLC-Q-Exactive Orbitrap MS metabonomic approach, which provided a deep understanding of the urate-lowering effects of polydatin. We screened 14 metabolites related to HUA (Table 1). Analysis of the metabolites showed that they were mainly involved in amino acid, lipid, and energy metabolism.

**FIGURE 7:**
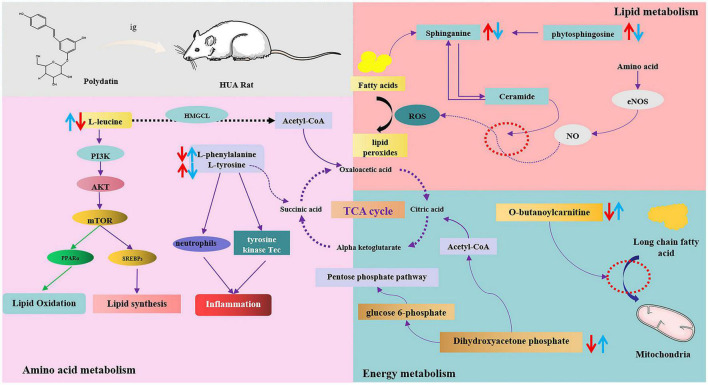
The intervention metabolisms of polydatin on HUA rats. The red arrow represents the changes of metabolites in hyperuricemic rats, and the blue arrow represents the changes of metabolites after oral administration of polydatin.

Moreover, these changes in hyperuricemic rats were reversed after oral administration of polydatin. Some possible mechanisms are summarized in Figure 7. The results indicated relevant metabolic pathways that were directly associated with HUA and proved that polydatin could ameliorate the related indicators, as discussed below.

(1) Hyperuricemia-induced changes in amino acid metabolism were reversed by polydatin.

Amino acids play important roles in vital activities, and amino acid metabolism regulates both protein and energy metabolism. The levels of L-leucine and L-phenylalanine decreased, whereas those of L-tyrosine significantly increased in HUA model rats (Figure 5B), indicating imbalances between protein and amino acid synthesis. These include phenylalanine, tyrosine, and tryptophan biosynthesis, phenylalanine metabolism, and aminoacyl-tRNA biosynthesis.

Leucine is a ketogenic amino acid that can be degraded to acetyl-CoA and acetoacetic acid in the body, entering glycolysis or other metabolic pathways through the tricarboxylic acid cycle, and participating in the regulation of the body’s normal metabolic processes. It has been shown that leucine is associated with the PIK3 and protein kinase C signaling pathways, which regulate glucose uptake and utilization in the cell and are related to insulin resistance and the development of type 2 diabetes (32). Leucine, valine, and isoleucine are branched-chain amino acids (BCAAs). BCAAs and their catabolic products participate in the regulation of numerous physiological processes in the body. Zhang et al. showed that leucine and isoleucine could be potential biomarkers for patients with HUA and gout (33). BCAAs are key factors in the development of metabolic diseases, and altered BCAAs may be indicators of type 2 diabetes mellitus (34, 35). However, since high UA levels are closely associated with the development of type 2 diabetes mellitus and other metabolic diseases, leucine may be a key indicator of HUA and other related diseases. In our study, after the administration of polydatin, the level of leucine reversed significantly compared with that of the untreated HUA group, implying that lowering UA by polydatin was related to the adjustment of background leucine levels.

The essential amino acids L-tyrosine and L-phenylalanine participate in phenylalanine, tyrosine, and tryptophan biosynthesis and phenylalanine metabolism. Phenylalanine can be hydroxylated to produce tyrosine by phenylalanine hydroxylase, which is involved in synthesizing essential neurotransmitters and hormones related to glucose and lipid metabolism (36). Increased tyrosine stimulated by monosodium urate (MSU) is correlated with the activation of neutrophils and tyrosine kinase Tec, leading to the release of inflammatory factors such as IL-1β, IL-8, IL-1, and tyrosine. It is thought to be a potential biomarker for MSU-induced gout (37). A study also showed that the level of tyrosine increased in acute gout in hyperuricemic rats (38).

Moreover, high UA levels cause the accumulation of MSU in the body, leading to the development of gouty arthritis, indicating that high tyrosine levels may be associated with the development of gout. Elevated tyrosine levels in the body can affect the functions of enzymes, such as citrate synthase, malate dehydrogenase, and succinate dehydrogenase, in the tricarboxylic acid cycle, leading to disturbances in energy metabolism and oxidative stress in the mitochondria (39). In our experiments, the level of tyrosine was elevated, whereas that of phenylalanine decreased in the model group, suggesting that overproduction of UA disturbed phenylalanine, tyrosine, and tryptophan biosynthesis and phenylalanine metabolism by accelerating the conversion of phenylalanine to tyrosine. However, polydatin decreased and increased the levels of tyrosine and phenylalanine, respectively. Phenylalanine, tyrosine, and tryptophan biosynthesis and phenylalanine metabolism were also altered by polydatin treatment compared with those in the untreated HUA group (Figure 6B), indicating that the restoration of a low UA level by polydatin involved regulation of phenylalanine and tyrosine metabolic pathways.

Aminoacyl-tRNA biosynthesis plays a vital role in the synthesis of proteins by recognizing the correct amino acids with tRNAs, including the corresponding anticodon for mRNA in the ribosome (40). Zhang et al. found that aminoacyl-tRNA biosynthesis was abnormal in patients with HUA and gout (33). Furthermore, aminoacyl-tRNA synthetases (AARSs) play an important role in aminoacyl-tRNA biosynthesis and have been verified in various human diseases (41). High UA levels may disturb aminoacyl-tRNA biosynthesis by acting on AARSs. In our experiment, aminoacyl-tRNA biosynthesis was restored after the oral administration of polydatin, suggesting that the mechanisms of polydatin may be associated with inhibiting AARSs’ activities. However, further studies are required to verify this hypothesis.

(2) Hyperuricemia-induced changes in lipid metabolism were reversed by polydatin.

Many researchers have reported that serum UA is associated with serum lipids in many diseases, such as HUA, gout, and cardiovascular and cerebrovascular diseases (33, 42). High serum TG levels were observed in a model of HUA (Figure 1). Moreover, a previous study showed that altered lipid metabolism is closely linked to HUA and gout (33). Our findings showed that lipid metabolism was disordered in hyperuricemic rats, which is consistent with the findings of previous studies, as discussed below.

Sphingolipids are amphiphilic lipids that include a sphingosine backbone and phospholipids, such as sphingol (Sph), ceramide (Cer), sphingomyelin (SM), gangliosides, and others. Intracellular sphingolipid metabolism plays a vital role in various metabolic diseases (43, 44). Sphinganine and phytosphingosine are involved in sphingolipid metabolism. Sphinganine can be converted into phytosphingosine by sphinganine C4-monooxygenase. Sphinganine and fatty acids of different chain lengths can form ceramides, which are central to sphingolipid synthesis and degradation. Studies have shown that ceramides mediate the conversion of NO to H_2_O_2_ in coronary microcirculation, thereby causing proinflammatory, prothrombotic, and atherogenic effects (45, 46). In this study, serum UA levels were elevated in HUA-model rats. The levels of sphinganine and phytosphingosine in the serum samples were elevated, which greatly promoted ceramide synthesis and caused abnormal sphingolipid metabolism *in vivo*. Therefore, abnormal sphingolipid metabolism may be the causative factor of cardiovascular disease caused by HUA. These trends were reversed compared to those found in normal rats after polydatin administration, indicating that polydatin might regulate sphingolipid metabolism to exert a UA-lowering effect.

(3) Hyperuricemia-induced changes in energy metabolism were reversed by polydatin.

Carnitine is essential for transporting long-chain fatty acids across the mitochondrial membrane. It also modulates the proportion of acyl-CoA/CoA and transfers some toxic acyl-CoA compounds. Derangement in the ability to transport long-chain fatty acids leads to the accumulation of harmful fatty acyl metabolites, which hinder gluconeogenesis and the citric acid cycle (47, 48). Therefore, carnitine is a powerful tool to identify whether energy metabolism is perturbed. Furthermore, carnitine transporters participate in the uptake and excretion of organic cations in the kidneys, and abnormal carnitine metabolism affects UA excretion in the kidneys (49). In this study, the level of *O*-butanoylcarnitine significantly decreased in the HUA model group. Treatment with polydatin resulted in a significant elevation in the levels of *O*-butanoylcarnitine, indicating that polydatin reversed the UA-induced disturbance of carnitine metabolism.

Dihydroxyacetone phosphate is an important component in gluconeogenesis and lipid metabolism. Dihydroxyacetone phosphate can be converted into glucose 6-phosphate and enter the gluconeogenic pathway. Moreover, dihydroxyacetone phosphate can be aerobically oxidized to produce acetyl CoA and enter the lipid metabolism pathway. The results showed that the level of dihydroxyacetone phosphate was decreased in hyperuricemic rats, indicating possible abnormalities in glucose and lipid metabolism. Aberrant dihydroxyacetone phosphate might be another important factor leading to changes in hyperuricemic rats’ blood glucose and triglyceride levels. However, polydatin caused dihydroxyacetone phosphate to return to normal levels, demonstrating good regulation of energy metabolism.

## 5. Conclusion

Polydatin is a functional compound derived from mulberries and grapes, with multiple bioactivities, including lowering the level of UA in serum and tissues. In this study, a UHPLC-Q-Exactive Orbitrap MS-based metabolomics approach was applied to explore the potential molecular mechanisms underlying the urate-lowering of polydatin in hyperuricemic rats. A total of 14 differential metabolites were identified in response to the therapeutic effects of polydatin, which were associated with amino acid metabolism, lipid metabolism, and energy metabolism. After polydatin intervention, the results showed that differential metabolites and the metabolic network were restored, approaching the levels observed in normal rats. This study has the potential to enhance our understanding of the mechanisms of HUA and demonstrate that polydatin is a promising adjuvant for lowering UA levels and alleviating HUA-related diseases.

## Data availability statement

The datasets presented in this study can be found in online repositories. The names of the repository/repositories and accession number(s) can be found in the article/Supplementary material.

## Ethics statement

All experimental protocols were approved by the Animal Ethics Committee of Binzhou Medical University (No. 2022-353).

## Author contributions

XG, XZ, and ZSh performed the experiments. PZ, KH, SZ, SH, YhW, YqW, and MZ analyzed the data. XL and ZSu wrote the manuscript. JZ conceived the project, designed the experiments, and revised the manuscript. All authors edited and approved the final version of the manuscript.

## Footnotes

1
https://hmdb.ca/

2
https://pubchem.ncbi.nlm.nih.gov/

3
http://www.metaboanalyst.ca

4
http://www.kegg.com/

## References

[1] Bardin T Richette P. Definition of hyperuricemia and gouty conditions. *Curr Opin Rheumatol.* (2014) 26:186–91. 10.1097/BOR.0000000000000028 24419750

[2] Ali N Perveen R Rahman S Mahmood S Rahman S Islam S. Prevalence of hyperuricemia and the relationship between serum uric acid and obesity: a study on bangladeshi adults. *PLoS One.* (2018) 13:e0206850. 10.1371/journal.pone.0206850 30383816 PMC6211757

[3] Yamanaka H. Gout and hyperuricemia in young people. *Curr Opin Rheumatol.* (2011) 23:156–60. 10.1097/BOR.0b013e3283432d35 21169841

[4] Li C Li Z Liu S Wang C Han L Cui L. Genome-wide association analysis identifies three new risk loci for gout arthritis in han Chinese. *Nat Commun.* (2015) 6:7041. 10.1038/ncomms8041 25967671 PMC4479022

[5] Yanai H Adachi H Hakoshima M Katsuyama H. Molecular biological and clinical understanding of the pathophysiology and treatments of hyperuricemia and its association with metabolic syndrome, cardiovascular diseases and chronic kidney disease. *Int J Mol Sci.* (2021) 22:9221. 10.3390/ijms22179221 34502127 PMC8431537

[6] Shao L Wei L. Efficacry and safety of benbromarne and allopurinol for primary gout ULT: a meta-analysis. *Chin J of Evid-Based Med.* (2012) 12:722–6. 28379501

[7] Imai S Nasuhara Y Momo K Oki H Kashiwagi H Sato Y. Implementation status of liver function tests for monitoring benzbromarone-induced hepatotoxicity: an epidemiological survey using the Japanese claims database. *Biol Pharm Bull.* (2021) 44:1499–505. 10.1248/bpb.b21-00393 34602558

[8] Childs L Dow C. Allopurinol-induced hepatomegaly. *BMJ Case Rep.* (2012) 2012:bcr2012007283. 10.1136/bcr-2012-007283 23087287 PMC4544329

[9] Stone RB. Lifting the veil on traditional Chinese medicine. *Science.* (2008) 319:709–10. 10.1126/science.319.5864.709 18258866

[10] Du QH Peng C Zhang H. Polydatin: a review of pharmacology and pharmacokinetics. *Pharm Biol.* (2013) 51:1347–54. 10.3109/13880209.2013.792849 23862567

[11] Oliviero F Zamudio-Cuevas Y Belluzzi E Andretto L Scanu A. Polydatin and resveratrol inhibit the inflammatory process induced by urate and pyrophosphate crystals in THP-1 cells. *Foods.* (2019) 8:560. 10.3390/foods8110560 31703439 PMC6915461

[12] Shi YW Wang CP Liu L Liu YL Wang X Hong Y. Antihyperuricemic and nephroprotective effects of resveratrol and its analogues in hyperuricemic mice. *Mol Nutr Food Res.* (2012) 56:1433–44. 10.1002/mnfr.201100828 22865646

[13] Wu G Wu HB Jiang H. [Anti-hyperuricemia effect and mechanism of polydatin in mice]. *Yao Xue Xue Bao.* (2014) 49:1739–42. 25920206

[14] Chen L Lan Z. Polydatin attenuates potassium oxonate-induced hyperuricemia and kidney inflammation by inhibiting NF-κB/NLRP3 inflammasome activation via the AMPK/SIRT1 pathway. *Food Funct.* (2017) 8:1785–92. 10.1039/C6FO01561A 28428988

[15] Hisamatsu T Okamoto S Hashimoto M Muramatsu T Andou A Uo M. Novel, objective, multivariate biomarkers composed of plasma amino acid profiles for the diagnosis and assessment of inflammatory bowel disease. *PLoS One.* (2012) 7:e31131. 10.1371/journal.pone.0031131 22303484 PMC3269436

[16] Lu X Xiong Z Li J Zheng S Huo T Li F. Metabonomic study on “kidney-yang deficiency syndrome” and intervention effects of rhizoma drynariae extracts in rats using ultra performance liquid chromatography coupled with mass spectrometry. *Talanta.* (2011) 83:700–8. 10.1016/j.talanta.2010.09.026 21147309

[17] Klassen A Faccio AT Canuto GA da Cruz PL Ribeiro HC Tavares MF. Metabolomics: definitions and significance in systems biology. *Adv Exp Med Biol.* (2017) 965:3–17. 10.1007/978-3-319-47656-8_1 28132174

[18] Zhao DS Jiang LL Wang LL Wu ZT Li ZQ Shi W. Integrated metabolomics and proteomics approach to identify metabolic abnormalities in rats with *Dioscorea bulbifera* rhizome-induced hepatotoxicity. *Chem Res Toxicol.* (2018) 31:843–51. 10.1021/acs.chemrestox.8b00066 30052031

[19] Souto-Carneiro M Tóth L Behnisch R Urbach K Klika KD Carvalho R. Differences in the serum metabolome and lipidome identify potential biomarkers for seronegative rheumatoid arthritis versus psoriatic arthritis. *Ann Rheum Dis.* (2020) 79:499–506. 10.1136/annrheumdis-2019-216374 32079570 PMC7147174

[20] Corona G Polesel J Fratino L Miolo G Rizzolio F Crivellari D. Metabolomics biomarkers of frailty in elderly breast cancer patients. *J Cell Physiol.* (2014) 229:898–902. 10.1002/jcp.24520 24659054

[21] Wang Y Liu Z Wang S Wang P Dong F Dai L. Effect of bidirectional fermentation system of paecilomyces cicadae/astragalus membranaceus in hyperuricemia models and its components. *Mod. Chin. Med.* (2020) 22:1638–43.

[22] Han B Gong M Li Z Qiu Y Zou Z. NMR-based metabonomic study Reveals intervention effects of polydatin on potassium oxonate-induced hyperuricemia in rats. *Oxid Med Cell Longev.* (2020) 2020:6943860. 10.1155/2020/6943860 32695259 PMC7362289

[23] Shi X Zhuang L Zhai Z He Y Sun E. Polydatin protects against gouty nephropathy by inhibiting renal tubular cell pyroptosis. *Int J Rheum Dis.* (2022) 2022:14463. 10.1111/1756-185X.14463 36328529

[24] Yang C Wan J Zheng D. Effects of qutan huoxue decotion on expressions of miR-27a, p38MAPKand AQP9 in nonalcoholic fatty liver disease rats. *Chin J Inf Tradit Chin Med.* (2021) 28:77–81.

[25] Kusonmano K Vongsangnak W Chumnanpuen P. Informatics for metabolomics. *Adv Exp Med Biol.* (2016) 939:91–115. 10.1007/978-981-10-1503-8_5 27807745

[26] Kim YM Heyman HM. Mass spectrometry-based metabolomics. *Methods Mol Biol.* (2018) 1775:107–18. 10.1007/978-1-4939-7804-5_10 29876813

[27] Jang C Chen L Rabinowitz JD. Metabolomics and isotope tracing. *Cell.* (2018) 173:822–37. 10.1016/j.cell.2018.03.055 29727671 PMC6034115

[28] Chong J Wishart DS Xia J. Using metaboanalyst 4.0 for comprehensive and integrative metabolomics data analysis. *Curr Protoc Bioinformatics.* (2019) 68:e86. 10.1002/cpbi.86 31756036

[29] Pang Z Chong J Zhou G de Lima Morais DA Chang L. MetaboAnalyst 5.0: narrowing the gap between raw spectra and functional insights. *Nucleic Acids Res.* (2021) 49:W388–96. 10.1093/nar/gkab382 34019663 PMC8265181

[30] Xia J Wishart DS. Using metaboanalyst 3.0 for comprehensive metabolomics aata analysis. *Curr Protoc Bioinform.* (2016) 55:14.10.1–14.10.91. 10.1002/cpbi.11 27603023

[31] Pang Z Zhou G Ewald J Chang L Hacariz O Basu N. Using metaboanalyst 5.0 for LC-HRMS spectra processing, multi-omics integration and covariate adjustment of global metabolomics data. *Nat Protoc.* (2022) 17:1735–61. 10.1038/s41596-022-00710-w 35715522

[32] Yoon MS. The emerging role of branched-chain amino acids in insulin resistance and metabolism. *Nutrients.* (2016) 8:405. 10.3390/nu8070405 27376324 PMC4963881

[33] Zhang Y Zhang H Chang D Guo F Pan H Yang Y. Metabolomics approach by 1H NMR spectroscopy of serum reveals progression axes for asymptomatic hyperuricemia and gout. *Arthritis Res Ther.* (2018) 20:111. 10.1186/s13075-018-1600-5 29871692 PMC5989453

[34] Wang SM Yang RY Wang M Ji FS Li HX Tang YM. Identification of serum metabolites associated with obesity and traditional risk factors for metabolic disease in Chinese adults. *Nutr Metab Cardiovasc Dis.* (2018) 28:112–8. 10.1016/j.numecd.2017.09.009 29122443

[35] Wang TJ Larson MG Vasan RS Cheng S Rhee EP McCabe E. Metabolite profiles and the risk of developing diabetes. *Nat Med.* (2011) 17:448–53. 10.1038/nm.2307 21423183 PMC3126616

[36] Korner J Cline GW Slifstein M Barba P Rayat GR Febres G. A role for foregut tyrosine metabolism in glucose tolerance. *Mol Metab.* (2019) 23:37–50. 10.1016/j.molmet.2019.02.008 30876866 PMC6479665

[37] Popa-Nita O Marois L Paré G Naccache PH. Crystal-induced neutrophil activation: X. proinflammatory role of the tyrosine kinase tec. *Arthr Rheum.* (2008) 58:1866–76. 10.1002/art.23801 18512796

[38] Chen W Liu Y Wei M Shi L Wu Y Liu Z. Studies on effect of ginkgo biloba L. leaves in acute gout with hyperuricemia model rats by using UPLC-ESI-Q-TOF/MS metabolomic approach. *RSC Adv.* (2017) 7:42964–72. 10.1039/C7RA08519B

[39] Ferreira GK Scaini G Carvalho-Silva M Gomes LM Borges LS Vieira JS. Effect of L-tyrosine in vitro and in vivo on energy metabolism parameters in brain and liver of young rats. *Neurotox Res.* (2013) 23:327–35. 10.1007/s12640-012-9345-4 22847184

[40] Ibba M Soll D. Aminoacyl-tRNA synthesis. *Annu Rev Biochem.* (2000) 69:617–50. 10.1146/annurev.biochem.69.1.617 10966471

[41] Sissler M González-Serrano LE Westhof E. Recent advances in mitochondrial aminoacyl-tRNA synthetases and disease. *Trends Mol Med.* (2017) 23:693–708. 10.1016/j.molmed.2017.06.002 28716624

[42] Tsouli SG Liberopoulos EN Mikhailidis DP Athyros VG Elisaf MS. Elevated serum uric acid levels in metabolic syndrome: an active component or an innocent bystander? *Metabolism.* (2006) 55:1293–301. 10.1016/j.metabol.2006.05.013 16979398

[43] Jiang XC Liu J. Sphingolipid metabolism and atherosclerosis. *Handb Exp Pharmacol.* (2013) 216:133–46. 10.1007/978-3-7091-1511-4_7 23563655

[44] Parveen F Bender D Law SH Mishra VK Chen CC Ke LY. Role of ceramidases in sphingolipid metabolism and human diseases. *Cells.* (2019) 8:1573. 10.3390/cells8121573 31817238 PMC6952831

[45] Freed JK Beyer AM LoGiudice JA Hockenberry JC Gutterman DD. Ceramide changes the mediator of flow-induced vasodilation from nitric oxide to hydrogen peroxide in the human microcirculation. *Circ Res.* (2014) 115:525–32. 10.1161/CIRCRESAHA.115.303881 24920698 PMC4640193

[46] Yu Z Peng Q Li S Hao H Deng J Meng L. Myriocin and d-PDMP ameliorate atherosclerosis in ApoE-/- mice via reducing lipid uptake and vascular inflammation. *Clin Sci.* (2020) 134:439–58. 10.1042/CS20191028 32091078

[47] Breningstall GN. Carnitine deficiency syndromes. *Pediatr Neurol.* (1990) 6:75–81. 10.1016/0887-8994(90)90037-2 2187442

[48] Evangeliou A Vlassopoulos D. Carnitine metabolism and deficit–when supplementation is necessary? *Curr Pharm Biotechnol.* (2003) 4:211–9. 10.2174/1389201033489829 12769764

[49] Wang Y Bi C Pang W Liu Y Yuan Y Zhao H. Plasma metabolic profiling analysis of gout party on acute gout arthritis rats based on UHPLC-Q-TOF/MS combined with multivariate statistical analysis. *Int J Mol Sci.* (2019) 20:5753. 10.3390/ijms20225753 31731809 PMC6888674

