# Supplementary material for: Identifying the intervention mechanisms of polydatin in hyperuricemia model rats by using UHPLC-Q-Exactive Orbitrap mass spectroscopy metabonomic approach
Source: Front Nutr. 2023 Apr 28;10:1117460. doi: 10.3389/fnut.2023.1117460 (PMC10176606; doi:10.3389/fnut.2023.1117460)
Supplement: Supplementary file 1 [file Data_Sheet_1.PDF]

Figure S1

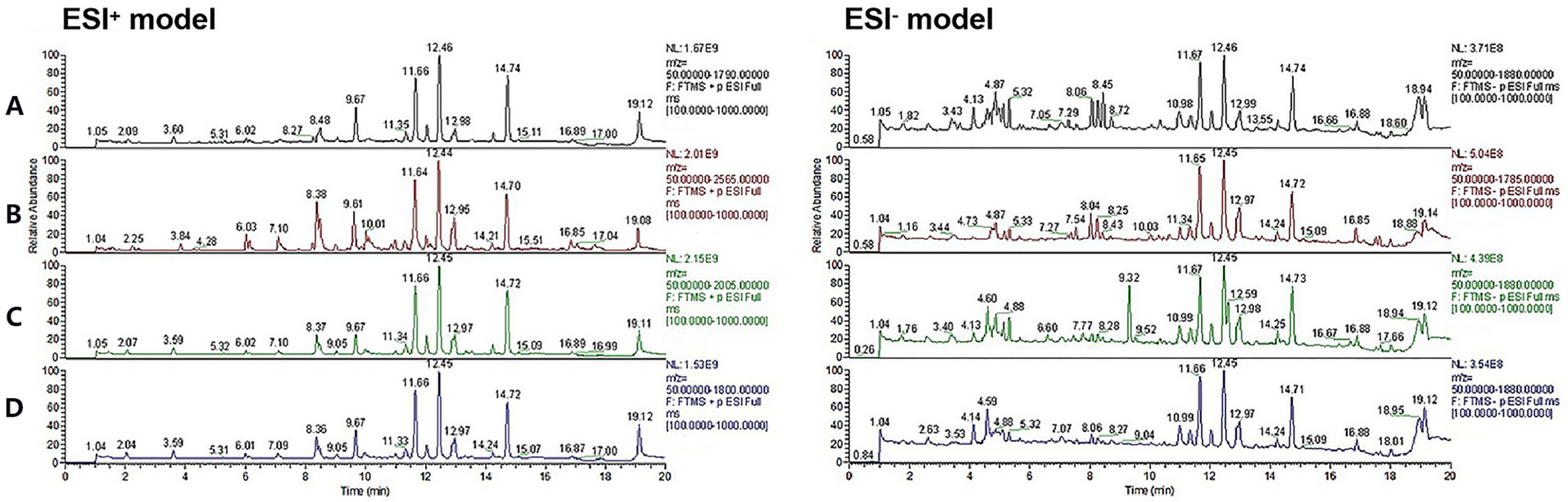

Figure S1: The typical serum base peak ion (BPI) flow diagram from ESI^+ mode and ESI^- mode in different groups (A: control group, B: model group, C: positive group, D: polydatin group).

Figure S2

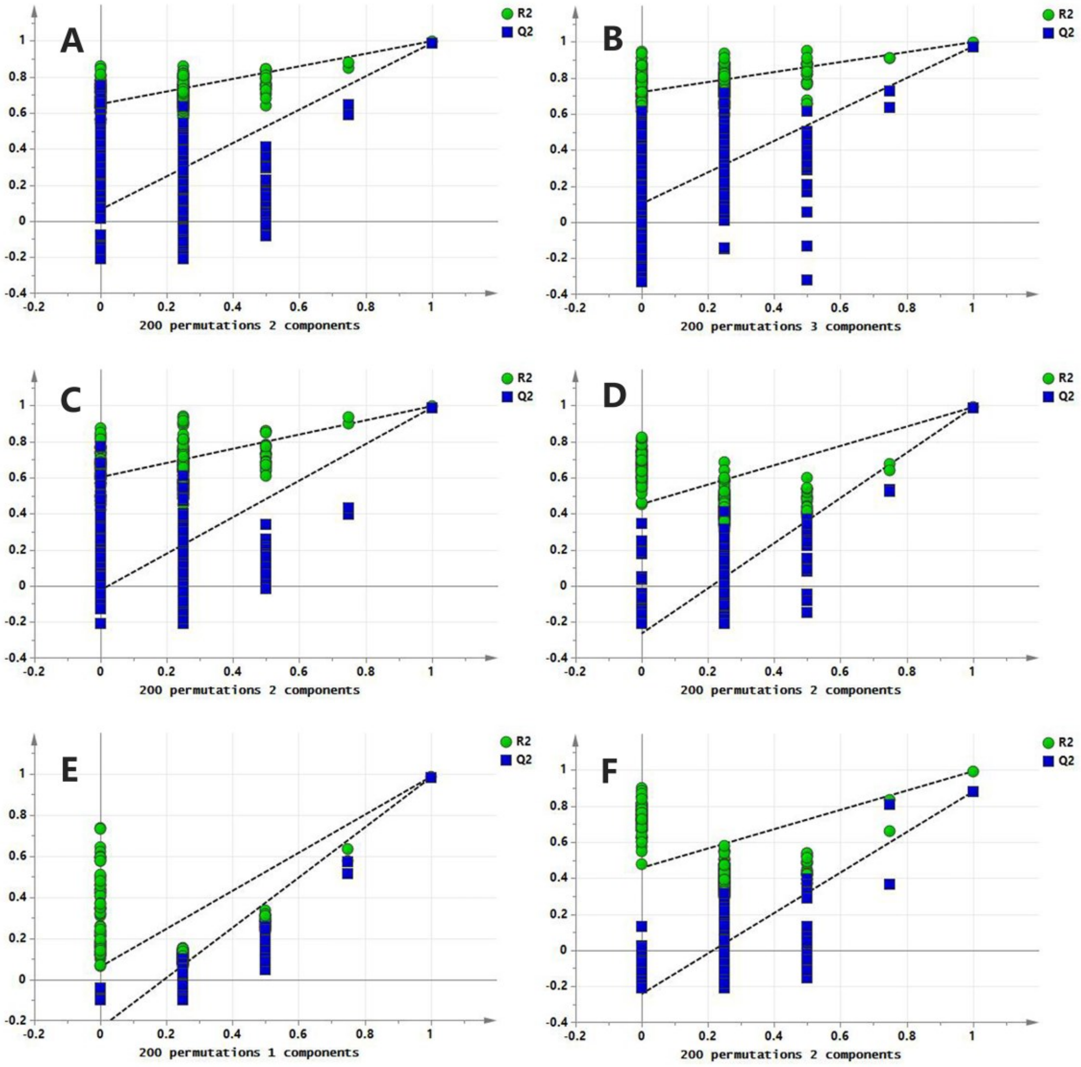

Figure S2: Permutation test plots of serum samples in different groups. Control and Model groups in ESI^+ mode (A) and ESI^- mode (B); Positive and Model groups in ESI^+ mode (C) and ESI^- mode (D); Polydatin and Model groups in ESI^+ mode (E) and ESI^- mode (F).

Figure S3

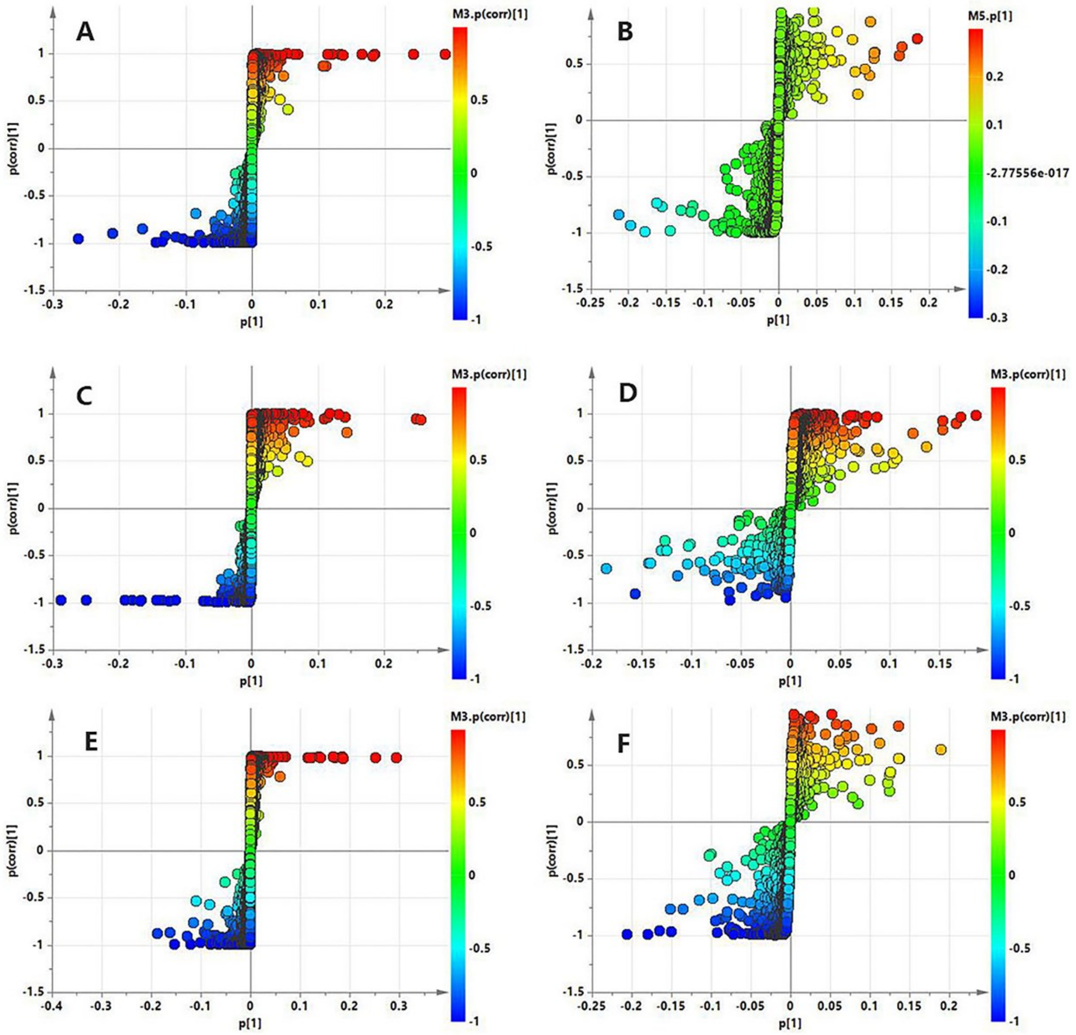

Figure S3: S-plot analysis of serum samples from the OPLS-DA model. Control and Model groups in ESI$^+$ mode (A) and ESI$^-$ mode (B); Positive and Model groups in ESI$^+$ mode (C) and ESI$^-$ mode (D); Polydatin and Model groups in ESI$^+$ mode (E) and ESI$^-$ mode (F)

Table S1 R^2Y and Q^2 values of serum samples from the OPLS-DA model

Samples	Ionic mode	group	R^2Y	Q^2
Serum	ESI^+	Control group and Model group	0.986	0.982
	ESI^+	Model group and Positive group	0.996	0.988
	ESI^+	Model group and Polydatin group	0.995	0.982
	ESI^-	Control group and Model group	0.998	0.987
	ESI^-	Model group and Positive group	0.990	0.980
	ESI^-	Model group and Polydatin group	0.993	0.975
